# Awareness of Community Members in Sudan Toward Stroke in 2024

**DOI:** 10.1002/puh2.70409

**Published:** 2026-10-14

**Authors:** Fatima Elbasri Abuelgasim Mohammed, Amna Alrasheed Abuzaid Ali, Ammar Elgadi, Ragad Magdi Mohammed Elamin Yousif, Israa Elbagir Mohamed Nassir, Ola Awad Babiker Adam, Reem Seifeldawla Taha Mohamed Osman, Igbal Izzeldin Ahmed Abuzeid, Yasir Eltayeb Elkhalifa Yousif Omer, Mohammed Alamin Mokhtar Omer, Rawan Seifeldawla Taha Mohamedosman, Wadaha Mohamed Nouh, Khalid Abbas Hassan Saad, Mohamed Eltag Abdelmutalib Mohamed, Khabab Abbasher Hussien Mohamed Ahmed

**Affiliations:** ^1^ Faculty of Medicine Khartoum University Khartoum Sudan; ^2^ Faculty of Medicine Al‐Neelain University Khartoum Sudan; ^3^ Faculty of Medicine Alzaiem Alazhari University Bahri Sudan; ^4^ Faculty of Medicine Alexandria University Alexandria Egypt; ^5^ Faculty of Medicine Bahri University Khartoum Sudan; ^6^ Faculty of Medicine Omdurman University Omdurman Sudan; ^7^ Department of Pathophysiology, School of Medicine St. George's University St. George's West Indies Grenada

**Keywords:** cerebrovascular accidents, community awareness, community health, risk factors, stroke

## Abstract

**Background:**

Sudan ranks 27th globally in stroke‐related deaths, with an age‐adjusted death rate of 136.47 per 100,000 people. Adequate knowledge among the population, particularly during times of conflict, can improve stroke outcomes.

**Objectives:**

To assess the Sudanese population's knowledge toward stroke in 2024.

**Methods:**

This cross‐sectional study was conducted from April to May 2024 among the Sudanese population who were aged more than 18 years. The study participants filled out a self‐administered online questionnaire that was composed of two sections: sociodemographic characteristics and knowledge toward stroke. The data were collected through convenience sampling. Frequencies, percentages, and associations were calculated through SPSS 28. Linear regression analysis was used to measure the association between the sociodemographic factors associated with stroke knowledge.

**Results:**

We received 1124 responses; 56.9% (*n* = 639) were female, and 80.8% (*n* = 908) were aged 18–36 years. High blood pressure (85.2%, *n* = 958) and previous history of stroke (72.3%, *n* = 813) were the most identified risk factors, whereas difficulty speaking (76.1%, *n* = 855) and severe unexplained headache (76.1%, *n* = 855) were the most recognized symptoms. However, 39.9% (*n* = 448), 46.6% (*n* = 524), and 43.3% (*n* = 487) did not recognize personality changes, depression, and bladder dysfunction, respectively, as possible complications of stroke.

**Conclusion:**

Awareness of stroke risk factors and the appropriate emergency response was generally adequate, but the recognition of warning symptoms and complications, such as personality changes, depression, and bladder dysfunction, was incomplete. As the sample was predominantly educated, awareness is likely lower in the general population, highlighting the need for targeted health‐education efforts on symptom and complication recognition, especially among less‐educated and rural groups.

## Graphical abstract

An online survey of 1124 Sudanese adults during armed conflict found moderate stroke awareness: Risk factors and emergency response were well recognized, but warning signs and complications were not. Strengthening public awareness is vital where emergency care is disrupted.

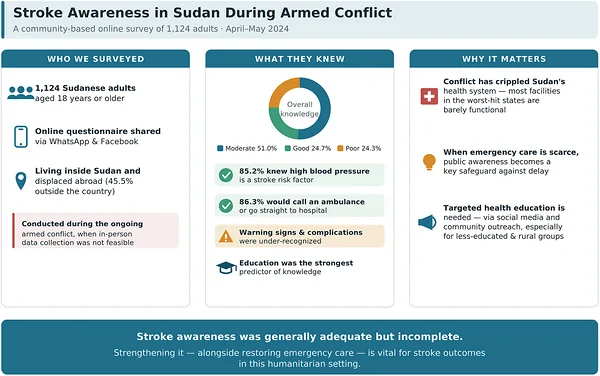

## 1 Introduction

Stroke, or cerebrovascular accident, is defined as an acute focal neurological deficit of the central nervous system resulting from hemorrhagic or ischemic causes [1]. Its occurrence is influenced by modifiable risk factors, such as high blood pressure, diabetes, heart disease, obesity, and smoking, as well as non‐modifiable factors, including age above 55 years, male sex (although females have a higher mortality rate), genetics, and a history of previous stroke [1]. Stroke mortality is also more common in low socioeconomic settings and during periods of extreme temperature [1].

In 2020, stroke was the second leading cause of death and third leading cause of disability worldwide, with the majority of cases occurring in low‐ and middle‐income countries where the burden is increasing more rapidly than in high‐income countries [1]. Africa has some of the highest stroke burdens in the world with an annual incidence of up to 316 per 100,000, prevalence as high as 1460 per 100,000 and a 3‐year fatality rate of over 80% [2]. Recent analyses have shown that stroke is among the leading causes of disability‐adjusted life years in African regions [2], and projections suggest that the incidence of stroke in Africa may be two to three times higher than in Western countries, mainly due to poor access to reliable medical information and poor control of risk factors [3, 4, 5]. In Sudan specifically, the WHO identifies stroke as the third leading cause of death [6], and the country ranks 27th globally in stroke‐related mortality, with an age‐adjusted death rate of 136.47 per 100,000 [7].

Community awareness of stroke—its warning signs, risk factors, and complications—is a key determinant of the success of both preventive action and timely treatment [5]. Poor awareness delays presentation to care and is associated with worse outcomes, as the effectiveness of acute stroke therapies is highly time‐dependent. Thus, an early recognition of symptoms allows timely and specialized intervention for the modification of disease course. Public education on risk factors, warning signs, and appropriate emergency response is critical to stroke prevention and control [9].

In Sudan, these challenges are further severely worsened by the armed conflict that began in April 2023. Beyond the preexisting vulnerability of a low‐resource population, the war has displaced more than 10 million people and led to the widespread destruction and closure of health facilities, crippling emergency and chronic‐disease services across much of the country [10]. By late 2024, the World Health Organization estimated that 70%–80% of health facilities in the worst‐affected states, and around 45% in other areas, were barely functional or closed, and it had verified more than 100 attacks on health care [11]. This creates a critical problem: The conflict simultaneously heightens stroke risk—through disrupted management of hypertension and diabetes, medication shortages, and chronic stress—while dismantling the health services required to treat it [12]. In such a context, where formal emergency systems are severely degraded, the ability of individuals and families to recognize stroke and seek care promptly becomes one of the few remaining safeguards, elevating the importance of community awareness even further. Yet, to our knowledge, the awareness of stroke among the Sudanese population during this humanitarian crisis has not been assessed.

Understanding the current level of awareness is a necessary first step for designing effective, context‐appropriate stroke education programs that can shorten the interval between symptom onset and the first emergency medical contact. Therefore, this study aimed to assess knowledge of stroke—including its risk factors, warning signs, complications, and appropriate emergency response—among the Sudanese population during the ongoing conflict.

## 2 Methodology

### 2.1 Study Design, Setting, Duration, and Participant

A cross‐sectional community‐based study was conducted from April to May 2024. Sudan is a country in northeast Africa, located between latitude 15°00 N and longitude 30°00 E, administratively divided into 18 states, with an estimated population of approximately 50 million [14]. Owing to the ongoing armed conflict that began in April 2023, in‐person data collection was not feasible; therefore, the study was conducted using a self‐administered, closed‐ended online questionnaire distributed through community groups and social media platforms, mainly WhatsApp and Facebook, which are widely used by Sudanese community members. The study targeted the general Sudanese population aged 18 years or older, recruited through convenience sampling.

### 2.2 Study Population and Eligibility Criteria

The target population was the general Sudanese population. Inclusion criteria were as follows: (1) Sudanese nationals aged 18 years or older, whether residing inside Sudan or displaced outside the country due to the conflict and (2) ability to read Arabic.

Sudanese nationals residing outside Sudan were included because the ongoing war has displaced a substantial proportion of the population across borders; these individuals remain members of the Sudanese community, and excluding them would have introduced selection bias against conflict‐affected groups. The same eligibility criteria were applied to them as to participants inside Sudan, and they were identified through the same Sudanese community groups and social media platforms used for recruitment.

### 2.3 Sample Size

The minimum required sample size was calculated using Cochran's formula for cross‐sectional studies with an unknown population size:

$$n=Z^{2}\times p\times \left(1-p\right)/d^{2}$$

where *Z* = 1.96 (the standard normal value at a 95% confidence level), *p* = 0.5 (assumed proportion of awareness, used to yield the maximum sample size), and *d* = 0.05 (margin of error). This gave a minimum required sample size of 385 participants. A total of 1124 participants were included in the final analysis.

### 2.4 Sampling Technique and Recruitment

Participants were recruited through non‐probability convenience sampling. A link to the questionnaire was circulated through Sudanese general community groups on WhatsApp and Facebook, the social media platforms most heavily used by the Sudanese population, and members were also encouraged to share the link within their networks. This approach was adopted because the conflict setting made probability‐based, in‐person sampling restricted.

### 2.5 Data Collection Instrument Development

Data were collected using a structured, self‐administered, and closed‐ended questionnaire adapted from validated instruments used in similar previous studies [15]. The questionnaire consisted of two sections: the first covered participants’ demographic characteristics, and the second assessed awareness of stroke risk factors, warning signs and symptoms, complications, and the first action participants would take if they experienced stroke symptoms. The questionnaire was translated to Arabic, the native language of the Sudanese population, using back‐translation by bilingual (native Arabic‐speaking, fluent English) professionals with a medical background to ensure semantic accuracy. Content validity was assessed through review by an academic expert in community medicine and neurology research, and the questionnaire was modified according to the feedback received. The instrument was then piloted on 20 individuals from the general population to assess clarity and reliability; Cronbach's alpha was 0.893, indicating high internal consistency. Responses from the pilot were not included in the final analysis.

### 2.6 Data Collection Procedure

Data were collected between April and May 2024 using Google Forms. The questionnaire link, accompanied by a brief description of the study's purpose, was distributed through Sudanese community groups on WhatsApp and Facebook. The first page of the form contained the participant information statement and electronic consent; only respondents who consented could proceed to the questionnaire. All questions were closed‐ended and marked as required to minimize missing data.

### 2.7 Data Analysis Plan

Data were analyzed using SPSS version 28. A knowledge score was computed by assigning one point for each correctly answered knowledge item across the four domains (general knowledge, risk factors, warning signs, and complications), giving a total score ranging from 0 to 36. Categorical variables were summarized as frequencies and percentages. The knowledge score, which was non‐normally distributed (on visual inspection), was summarized as median with first and third quartiles. Bivariate associations between the knowledge score and demographic variables were tested using the Mann–Whitney *U* test for two‐category variables and the Kruskal–Wallis test for variables with more than two categories. Multivariable linear regression was then used to identify independent predictors of the knowledge score; the dependent variable was the total knowledge score and its sub‐scores for the domain‐specific models. The assumptions of normality and homoscedasticity were assessed using Shapiro–Wilk and Levene's tests, respectively. Multicollinearity among the predictors was checked using the variance inflation factor (VIF). Given the large sample size (*n* = 1124), linear regression was considered robust to the non‐normality of the raw score. A *p* value <0.05 was considered statistically significant.

## 3 Results

### 3.1 Sociodemographic Characteristics of Participants

A total of 1124 participants were included. Females represented 56.9% (*n* = 639) and males 43.1% (*n* = 485). Most participants were young adults aged 18–36 years (80.8%, *n* = 908), and the majority were single (64.8%, *n* = 728). Nearly half (45.5%, *n* = 511) resided outside Sudan due to the ongoing war. Regarding education, 70.2% (*n* = 789) held university degrees, and 17.2% (*n* = 193) held postgraduate degrees. More than half (56.9%, *n* = 640) had no health insurance, and most were nonsmokers (82.4%, *n* = 926). These findings are summarized in Table 1.

**TABLE 1:** Demographic characteristics of the study participants.

		*n*	%
Gender	Female	639	56.9
	Male	485	43.1
Age	18–36	908	80.8
	37–55	179	15.9
	56–74	35	3.1
	75 and more	2	0.2
Marital status	Single	728	64.8
	Engaged	88	7.8
	Married	276	24.6
	Divorced	27	2.4
	Widow	5	0.4
Place of residence	Outside Sudan due to the war	511	45.5
	Khartoum	104	9.3
	Gezira	29	2.6
	Red Sea	106	9.4
	Kassala	64	5.7
	Gadaref	48	4.3
	Sennar	24	2.1
	White Nile	44	3.9
	Blue Nile	18	1.6
	Northern	74	6.6
	River Nile	96	8.5
	North Kordofan	2	0.2
	West Kordofan	1	0.1
	South Darfur	3	0.3
Educational level	Primary school	4	0.4
	Intermediate school	8	0.7
	Secondary school	130	11.6
	University	789	70.2
	Postgraduate degree	193	17.2
Do you have health insurance	No	640	56.9
	Yes	484	43.1
Do you smoke: cigarettes, hookah, and so on	No	926	82.4
	Yes	198	17.6

### 3.2 Knowledge Toward Stroke

Most respondents correctly identified that stroke is not an infectious disease (96.3%, *n* = 1082) and can be prevented (82.2%, *n* = 924). Only 13% (*n* = 146) believed that stroke affects only the elderly, and 18.9% (*n* = 212) considered it hereditary. The most identified risk factors were high blood pressure (85.2%, *n* = 958), previous history of stroke (72.3%, *n* = 813), heart disease (69.2%, *n* = 778), atherosclerosis (65.2%, *n* = 733), and high lipids (65.0%, *n* = 731), whereas stress (11.7%, *n* = 132) and gender (21.7%, *n* = 244) were the least identified. The most recognized symptoms were difficulty speaking (76.1%, *n* = 855), severe unexplained headache (76.1%, *n* = 855), dizziness, difficulty walking, imbalance, and uncoordinated movements (each 72.6%, *n* = 816), and blurred vision (69.0%, *n* = 776); shortness of breath (31.2%, *n* = 351) and chest pain/anxiety (33.6%, *n* = 378) were the least recognized. Regarding complications, most participants recognized paralysis and movement problems (85.4%, *n* = 960), memory and cognition problems (78.3%, *n* = 880), and blurred vision (75.2%, *n* = 845), whereas depression (53.4%, *n* = 600) and personality changes (60.1%, *n* = 676) were recognized less often. These findings are presented in Table 2.

**TABLE 2:** Participants’ knowledge toward stroke.

	Yes		No	
	*n*	%	*n*	%
Stroke is an infectious disease	42	3.7	1082	96.3
Stroke affects only the elderly	146	13	978	87
Stroke is a hereditary disease	212	18.9	912	81.1
Stroke can be prevented	924	82.2	200	17.8
**Stroke risk factors**				
High blood pressure	958	85.2	166	14.8
High lipids	731	65.0	393	35.0
Diabetes	655	58.3	469	41.7
Heart diseases	778	69.2	346	30.8
Unhealthy lifestyle	729	64.9	395	35.1
Drinking alcohol	638	56.8	486	43.2
Smoking	678	60.3	446	39.7
History of previous stroke	813	72.3	311	27.7
Genetic factors	437	38.9	687	61.1
Obesity	567	50.4	557	49.6
Age as risk factor	560	49.8	564	50.2
Atherosclerosis	733	65.2	391	34.8
Blood viscosity	466	41.5	658	58.5
Lack of exercise	485	43.1	639	56.9
Gender	244	21.7	880	78.3
Stress	132	11.7	992	88.3
**Stroke symptoms**				
Difficulty speaking or expressing or understanding	855	76.1	269	23.9
Blurred vision in one or both eyes	776	69.0	348	31.0
Dizziness	816	72.6	308	27.4
Difficulty walking	816	72.6	308	27.4
Imbalance	816	72.6	308	27.4
Uncoordinated movements	816	72.6	308	27.4
Numbness or weakness on one side of the face or limb	730	64.9	394	35.1
Shortness of breath	351	31.2	773	68.8
Severe unexplained headache	855	76.1	269	23.9
Chest pain/panic and anxiety	378	33.6	746	66.4
**Stroke complications**				
Personality changes may occur due to stroke	676	60.1	448	39.9
Blurred vision may occur due to stroke	845	75.2	279	24.8
Paralysis and movement problems may occur due to stroke	960	85.4	164	14.6
Memory and cognition problems may occur due to stroke	880	78.3	244	21.7
Depression may occur after stroke	600	53.4	524	46.6
Bladder and urinary control problems may occur due to stroke	637	56.7	487	43.3

### 3.3 Help‐Seeking Behavior for Stroke Symptoms

When asked about the first action they would take in response to stroke symptoms, 44.6% (*n* = 501) reported that they would call an ambulance and 41.7% (*n* = 469) would go directly to the hospital. Smaller proportions would consult a doctor (9.5%, *n* = 107), take medication (3.3%, *n* = 37), or wait and see (0.9%, *n* = 10). This pattern suggests generally adequate awareness of emergency response behavior (Figure 1).

**FIGURE 1:**
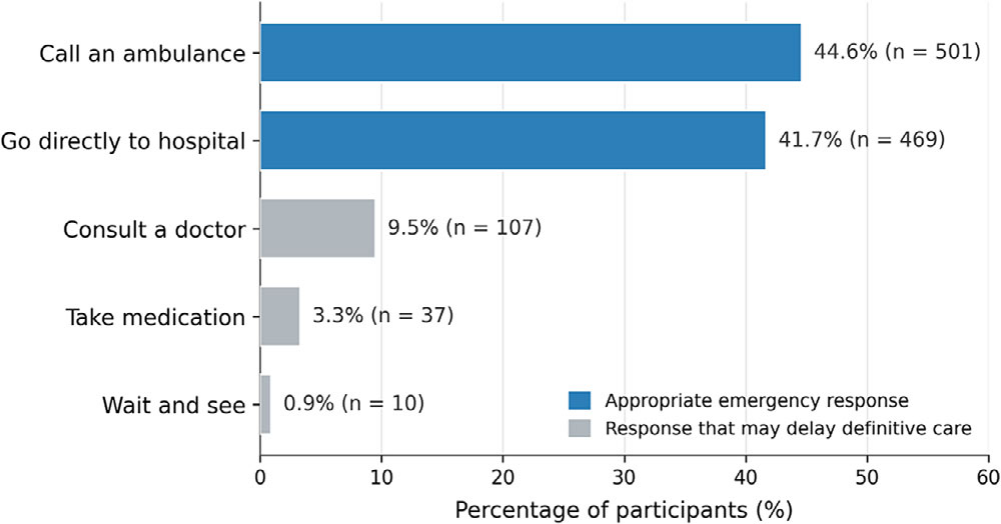
Help‐seeking behavior for stroke symptoms among the study participants (*n* = 1124).

### 3.4 Knowledge Score

The median overall stroke knowledge score was 23.00 (*Q*1 = 17.00, *Q*3 = 28.00). On the basis of percentile classification, 24.3% of participants demonstrated poor knowledge, 51.0% had moderate knowledge, and 24.7% exhibited good knowledge levels. This distribution indicates that the majority of the population possesses moderate awareness regarding stroke (Table 3).

**TABLE 3:** Association between stroke knowledge and the various demographic variables.

		Overall total score				
		Mdn	*Q*1	*Q*3	Test	*p*
Gender	Female	23.00	17.00	29.00	151,739.50^b^	0.55
	Male	22.00	16.00	28.00		
Age	18–36	23.00	17.00	29.00	4.286491^a^	0.23
	37–55	21.00	15.00	27.00		
	56–74	23.00	16.00	28.00		
	75 and more	18.00	2.00	34.00		
Marital status	Single	23.00	17.00	29.00	5.554^a^	0.24
	Engaged	23.00	18.00	27.00		
	Married	21.00	16.00	28.00		
	Divorced	23.00	14.00	28.00		
	Widow	17.00	13.00	17.00		
Place of residence	Outside Sudan	23.00	17.00	30.00	21.900^a^	0.06
	Khartoum	21.00	16.50	26.50		
	Gezira	18.00	13.00	27.00		
	Red Sea	22.00	17.00	26.00		
	Kassala	23.00	17.00	28.50		
	Gadaref	22.50	18.00	28.50		
	Sennar	21.50	17.50	25.50		
	White Nile	21.00	14.00	26.00		
	Blue Nile	17.00	10.00	24.00		
	Northern	24.50	20.00	30.00		
	River Nile	22.50	16.50	29.00		
	North Kordofan	16.50	16.00	17.00		
	West Kordofan	18.00	18.00	18.00		
	South Darfur	22.00	16.00	31.00		
Educational level	Primary school	10.00	2.50	21.50	51.986^a^	<0.01
	Intermediate school	18.50	15.50	23.00		
	Secondary school	20.00	14.00	24.00		
	University	23.00	16.00	28.00		
	Postgraduate degree	26.00	20.00	31.00		
Do you have health insurance	No	22.00	16.00	29.00	157,581.50^b^	0.62
	Yes	23.00	18.00	28.00		
Do you smoke: cigarettes, hookah, and so on	No	23.00	17.00	29.00	85,150.50^b^	0.12
	Yes	22.00	17.00	27.00		

^a^ Kruskal–Wallis test.

^b^ Mann–Whitney *U* test.

### 3.5 Association Between Knowledge and Demographic Variables

Statistical analysis using Kruskal–Wallis and Mann–Whitney *U* tests revealed no significant differences in stroke knowledge across gender, age, marital status, or region (*p* > 0.05). However, education level was significantly associated with better stroke knowledge (*p* < 0.01). Participants with postgraduate degrees showed the highest knowledge scores, emphasizing the role of education in promoting awareness (Table 3).

### 3.6 Predictors of Good Knowledge of Stroke

Linear regression analysis demonstrated that educational level (*β* = 0.231, *p* < 0.001) and health‐insurance status (*β* = 0.058, *p* = 0.049) were significant predictors of good stroke knowledge, whereas other variables were not significant (Figure 2).

**FIGURE 2:**
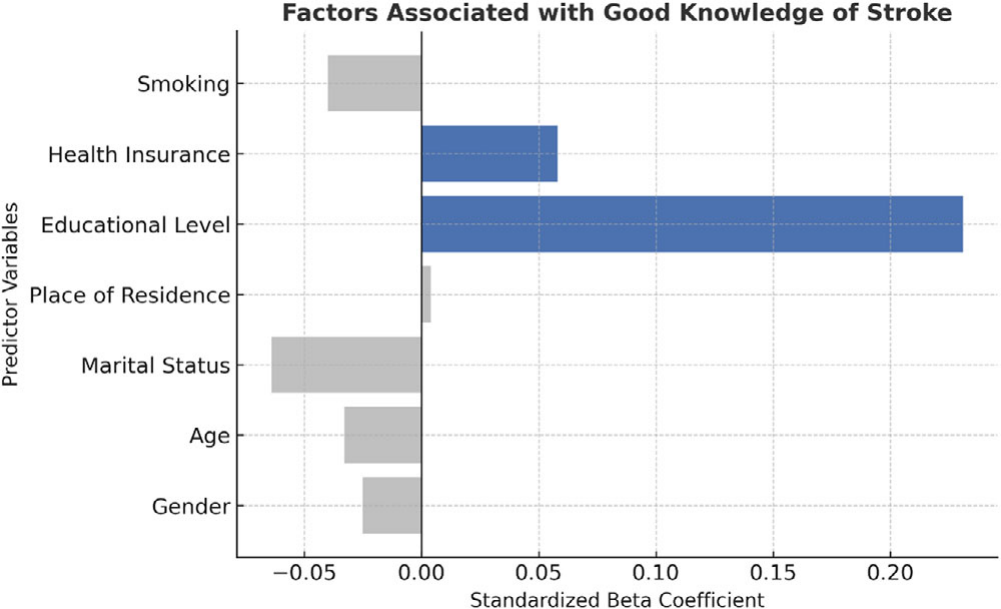
Linear regression analysis showing factors associated with *good knowledge of stroke* among the Sudanese population. Educational level and having health insurance were significant predictors (*p* < 0.05).

### 3.7 Predictors of General Knowledge

Educational level (*β* = 0.263, *p* < 0.001), gender (*β* = −0.072, *p* = 0.022), marital status (*β* = −0.112, *p* = 0.002), place of residence (*β* = −0.076, *p* = 0.008), health insurance (*β* = −0.060, *p* = 0.035), and smoking (*β* = −0.075, *p* = 0.015) were all significantly associated with general stroke knowledge, indicating that both socioeconomic and lifestyle factors influence awareness (Figure 3).

**FIGURE 3:**
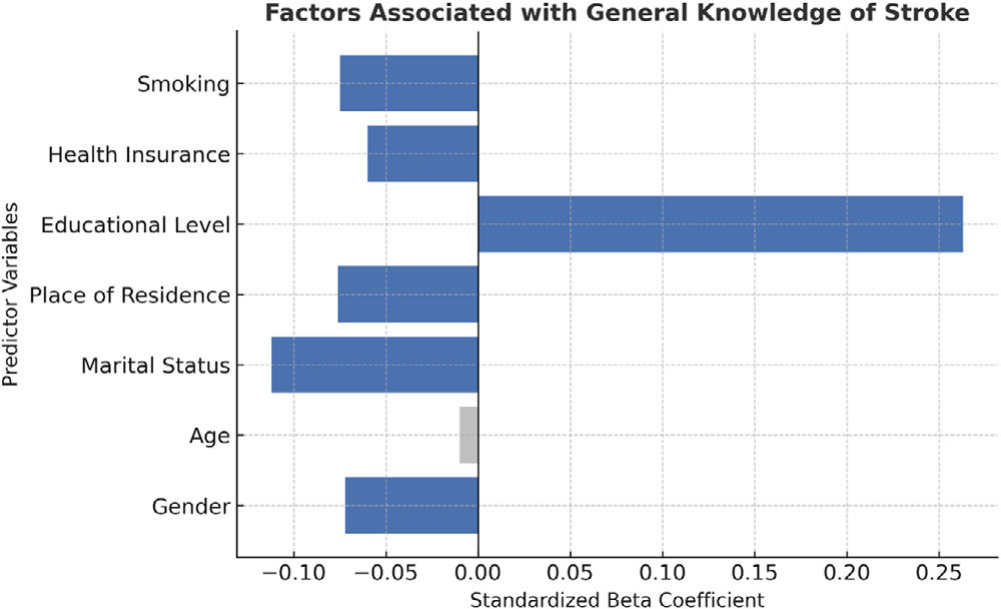
Linear regression analysis of factors influencing *general knowledge of stroke*. Significant predictors included educational level, gender, marital status, place of residence, health insurance, and smoking habits (*p* < 0.05).

### 3.8 Predictors of Knowledge of Risk Factors

Educational level was the only significant determinant of knowledge about stroke risk factors (*β* = 0.172, *p* < 0.001). Other factors, such as gender, age, marital status, and smoking habits, showed no statistical significance (Figure 4).

**FIGURE 4:**
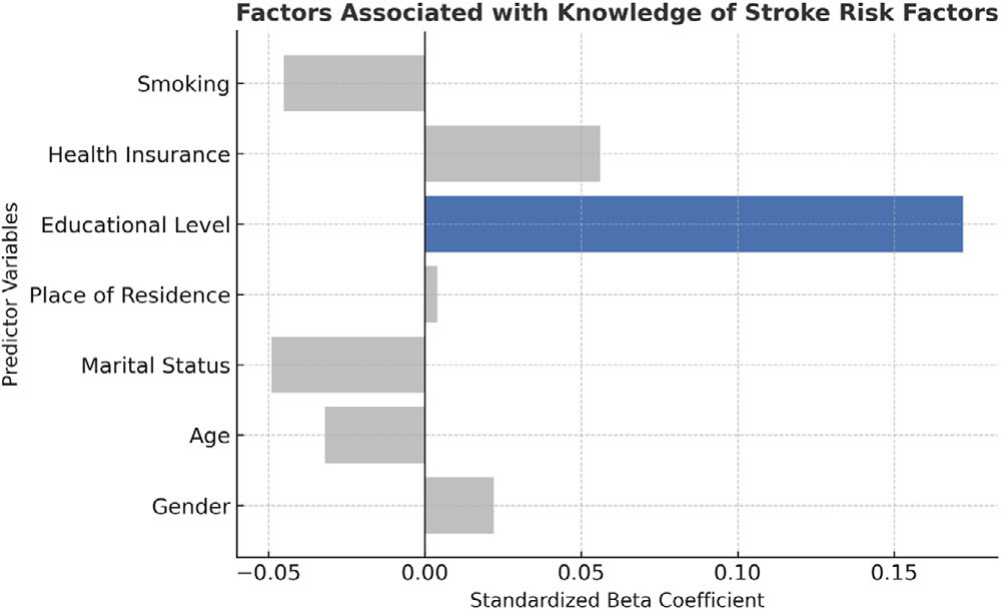
Linear regression analysis showing factors associated with *knowledge of stroke risk factors*. Only educational level was a significant predictor (*p* < 0.05).

### 3.9 Predictors of Knowledge of Stroke Signs

Both educational level (*β* = 0.131, *p* < 0.001) and health insurance (*β* = 0.075, *p* = 0.013) were found to significantly predict knowledge of stroke signs. This suggests that individuals with higher education and access to health‐care systems are more capable of recognizing early stroke symptoms (Figure 5).

**FIGURE 5:**
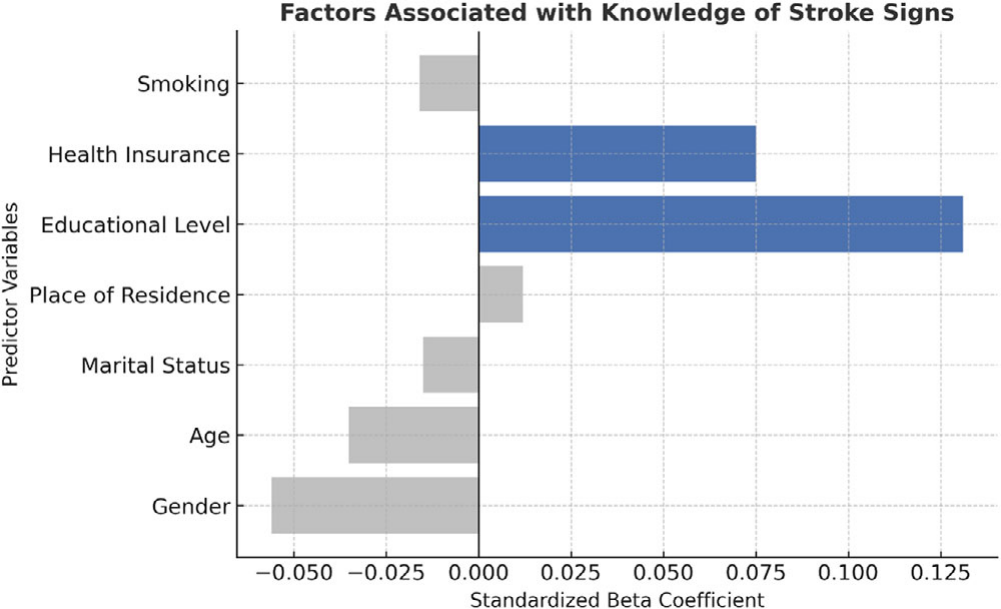
Linear regression analysis showing factors associated with *knowledge of stroke signs*. Educational level and having health insurance were significant predictors (*p* < 0.05).

### 3.10 Predictors of Knowledge of Stroke Complications

Finally, educational level (*β* = 0.235, *p* < 0.001) emerged as the sole significant predictor of awareness regarding stroke complications. Other demographic or lifestyle variables were not associated with this knowledge domain (Figure 6).

**FIGURE 6:**
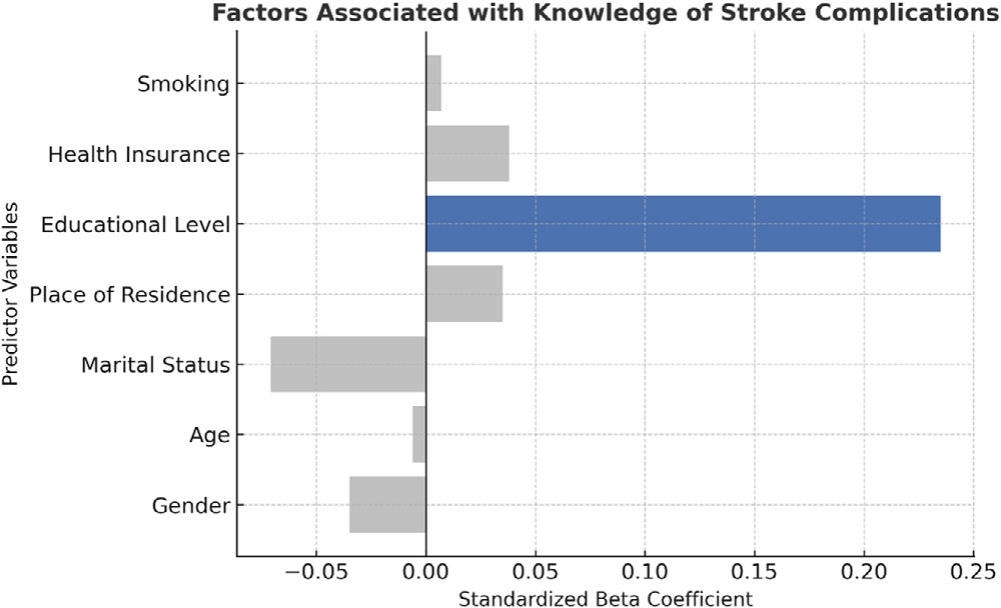
Linear regression analysis showing factors associated with *knowledge of stroke complications*. Educational level was the only significant predictor (*p* < 0.05).

## 4 Discussion

This community‐based study assessed stroke awareness among Sudanese adults during an active armed conflict, a setting in which a timely recognition of and response to stroke are especially consequential. Overall, participants demonstrated moderate knowledge (moderate 51.0%, good 24.7%, and poor 24.3%), with educational attainment emerging as the strongest predictor. This should be interpreted cautiously, however, as the sample was disproportionately young and highly educated (87.4% with a university or postgraduate degree) and, therefore, likely reflects a more health‐literate segment than the general Sudanese population.

High blood pressure was the most frequently recognized risk factor (85.2%), consistent with an earlier Sudanese study that identified hypertension [16] and with Nigerian studies recognizing hypertension and smoking [17]. Recognition in our sample was lower than the 99.8% reported in the United Arab Emirates (UAE) [18] and comparable to the 85.4% reported in Lebanon [19], differences that may reflect the higher educational and health‐system development of those settings. Notably, awareness that psychological stress (11.7%) and gender (21.7%) can influence stroke risk was low; by contrast, 80% of participants in the Lebanese study recognized psychological stress as a risk factor [19], suggesting the link between mental and physical health is underappreciated among our participants.

Our findings should also be read against the wider sub‐Saharan African context, where stroke awareness is generally low despite the region bearing a growing share of the global stroke burden. A population survey in Ghana found that fewer than half of respondents could recognize any established stroke risk factor or warning sign [20], and a household study in Accra similarly reported that only about 40% identified hypertension as a risk factor [21]. The comparatively higher recognition in our sample most likely reflects its educational profile rather than broadly higher awareness in Sudan, reinforcing the need for cautious interpretation.

The recognition of warning symptoms was strongest for difficulty speaking and severe unexplained headache (both 76.1%), followed by dizziness, imbalance, and difficulty walking (72.6%). This resembles the UAE study, in which difficulty speaking was the most commonly recognized symptom [18], whereas a Nigerian study found one‐sided weakness to be most recognized [22]. Participants were least likely to endorse shortness of breath (31.2%), chest pain, and panic or anxiety (33.6%); as these are nonclassical, less specific features, their lower endorsement may reflect uncertainty in distinguishing stroke from other acute conditions rather than a knowledge deficit.

Regarding help‐seeking behavior, most participants reported an appropriate emergency response, with 44.6% choosing to call an ambulance and 41.7% to go directly to a hospital (86.3% combined), whereas smaller proportions would consult a doctor (9.5%), take medication (3.3%), or wait and see (0.9%). This compares with 71% who would attend hospital in India [23], and 88% of French and 75% of American respondents who would call emergency services [24], reflecting the more established emergency systems in high‐income settings.

Across our analyses, educational attainment stood out as the dominant and most consistent driver of stroke knowledge, being the only factor independently linked to awareness of both risk factors and complications. Other sociodemographic characteristics—gender, marital status, place of residence, health insurance, and smoking—appeared to shape only broader, general knowledge rather than these specific domains, and health insurance seemed particularly relevant to recognizing warning signs, perhaps reflecting greater prior contact with the health system. Notably, age was not associated with knowledge, suggesting that awareness in this population is shaped more by education and access than by life stage. This central role of education mirrors findings elsewhere: In the UAE, women and more educated individuals demonstrated higher stroke knowledge [18], and an Iranian study similarly identified education as a key correlate of awareness of stroke risk factors and warning symptoms [25].

The findings of this study carry particular weight given the setting in which it was conducted. Sudan has been affected by armed conflict since April 2023, and 45.5% of our participants had been displaced outside the country. The conflict has devastated the health system: By late 2024, the World Health Organization estimated that 70%–80% of health facilities in the worst affected states, and around 45% elsewhere, were barely functional or closed, and it had verified more than 100 attacks on health care [11]. Emergency services, intensive care, and chronic‐disease management, including dialysis, have been widely disrupted, and shortages of medicines and the exodus of health workers have compounded the collapse [12]. This creates a dangerous paradox for stroke: Conflict raises risk—through interrupted management of hypertension and diabetes, medication shortages, poor nutrition, and chronic psychological stress—while simultaneously eroding the very services needed to treat it.

In such a setting, public awareness assumes greater importance than in stable contexts, because early recognition by patients and families and prompt care‐seeking may be among the few remaining safeguards when formal systems are degraded. The relatively adequate awareness observed here is therefore encouraging, but its value is conditional: Knowing to call an ambulance offers limited benefit where ambulances and functioning hospitals are scarce. These findings also point to a practical opportunity. Our questionnaire reached both internally located and displaced Sudanese through social media, demonstrating that digital platforms and diaspora networks are effective, low‐cost channels for health messaging when in‐person outreach is not feasible. Awareness interventions delivered through these channels should be embedded within wider efforts to restore emergency and primary care, so that improved recognition can translate into timely treatment.

### 4.1 Limitations

This study has several limitations. Its cross‐sectional design precludes causal inference. The online, social media–based convenience sampling, necessitated by the conflict, introduced selection bias toward younger, urban, internet‐connected, and highly educated participants, so the reported awareness levels likely overestimate those of the general Sudanese population, particularly older, rural, and less‐educated groups. As the questionnaire was self‐administered in Arabic online, individuals without literacy or internet access—including those in the most conflict‐affected areas—may have been underrepresented. Responses reflected intended rather than actual behavior and may be subject to social‐desirability bias. Finally, instrument validation relied on review by a single academic expert and back‐translation, which may not fully capture content validity.

### 4.2 Recommendations

Targeted health‐education campaigns should prioritize populations with lower educational attainment, rural communities, and school‐aged students and should emphasize the recognition of stroke warning signs, the long‐term complications of stroke, and the urgency of immediate care, using simple tools, such as the FAST message (Face, Arm, Speech, and Time). Given their demonstrated reach, these campaigns should combine social media and diaspora networks with in‐person outreach in remote and underserved areas. In the current humanitarian setting, such efforts should be embedded within wider work to restore emergency and primary‐care services so that improved recognition can translate into timely treatment. Future population‐based studies using representative sampling, and including less‐educated and rural groups, are needed to confirm these findings.

## 5 Conclusion

Awareness of stroke among the Sudanese participants was generally adequate, with good recognition of major risk factors and appropriate emergency response; most participants (86.3%) reported they would call an ambulance or go directly to a hospital if they experienced stroke symptoms. However, important gaps remained in recognizing warning symptoms and long‐term complications, and because the sample was predominantly young and highly educated, these findings likely overestimate awareness in the general population. Targeted health‐education efforts, delivered through both social media and in‐person outreach and paired with the restoration of emergency care in this conflict setting, are therefore needed to close these gaps—particularly among less‐educated and rural groups.

## Author Contributions

**Fatima Elbasri Abuelgasim Mohammed**: investigation, leadership, validation, writing of first draft, revision and writing of final draft. **Amna Alrasheed Abuzaid Ali**: project co‐leadership, validation, writing of first draft. **Ammar Elgadi**: data analysis and manuscript writing. **Ragad Magdi Mohammed Elamin Yousif**: data collection and manuscript first draft writing (equal). **Israa Elbagir Mohamed Nassir**: data collection and manuscript first draft writing (equal). **Ola Awad Babiker Adam**: data collection and manuscript first draft writing (equal). **Reem Seifeldawla Taha Mohamed Osman**: data collection and manuscript first draft writing (equal). **Igbal Izzeldin Ahmed Abuzeid**: data collection and manuscript first draft writing (equal). **Yasir Eltayeb Elkhalifa Yousif Omer**: data collection and manuscript first draft writing (equal). **Mohammed Alamin Mokhtar Omer**: data collection and manuscript first draft writing (equal). **Khalid Abbas Hassan Saad**: data collection and manuscript first draft writing (equal). **Wadaha Mohamed Nouh**: data collection and manuscript first draft writing (equal). **Rawan Seifeldawla Taha Mohamedosman**: data collection and manuscript first draft writing (equal). **Mohamed Eltag Abdelmutalib Mohamed**: data collection and manuscript first draft writing (equal). **Khabab Abbasher Hussien Mohamed Ahmed**: manuscript writing and revision. All authors reviewed the final version of the manuscript.

## Funding

The authors have nothing to report.

## Ethics Statement

Ethical approval was obtained from Nafeer Alafia Hospital, Northern State Ministry of Health, Sudan (Approval No. 200102). All methods were performed in accordance with the relevant guidelines and regulations. The study was conducted according to the principles and guidelines of Declaration of Helsinki.

## Consent

A fully informed consent was obtained from the participants prior to their participation. The consent was obtained electronically after the study objectives were explained to them.

## Conflicts of Interest

The authors declare no conflicts of interest.

## Data Availability

The data are provided by the corresponding author upon reasonable request.
